# Pharmacological Mechanism of *Aucklandiae Radix* against Gastric Ulcer Based on Network Pharmacology and In Vivo Experiment

**DOI:** 10.3390/medicina59040666

**Published:** 2023-03-27

**Authors:** Lan Feng, Lisha A, Huifang Li, Xiyele Mu, Na Ta, Laxinamujila Bai, Minghai Fu, Yongsheng Chen

**Affiliations:** 1 NMPA Key Laboratory for Quality Control of Traditional Chinese Medicine (Mongolian Medicine), School of Mongolian Medicine, Inner Mongolia Minzu University, Tongliao 028000, China; 2 Key Laboratory of Tropical Translational Medicine of Ministry of Education, Hainan Provincial Key Laboratory for Research and Development of Tropical Herbs, School of Pharmacy, Hainan Medical University, Haikou 571199, China

**Keywords:** *Aucklandiae Radix*, gastric ulcer, network analysis, molecular docking, pharmacological mechanism

## Abstract

*Background and Objectives*: *Aucklandiae Radix* is a well-known medicinal herb that is often used to treat gastric ulcer, but its molecular mechanism of anti-ulcer action is poorly understood. This research aimed to reveal the potential active components, core targets, and mechanisms of *Aucklandiae Radix* in treating gastric ulcer by combining network pharmacology and animal experimentation. *Materials and Methods*: First, a network pharmacology strategy was used to predict the main components, candidate targets, and potential signaling pathways. Molecular docking was then used to confirm the binding affinity between the main components and primary targets. Finally, rats were treated with indomethacin 30 mg/kg to establish a gastric ulcer model. *Aucklandiae Radix* extract (0.15, 0.3, and 0.6 g/kg) was pre-treated in rats by oral gavage for 14 days, and the protective effect and candidate targets of network pharmacology were validated through morphological observation, pathological staining, and biochemical index detection. *Results*: A total of eight potential active components and 331 predicted targets were screened from *Aucklandiae Radix*, 37 of which were common targets with gastric ulcer. According to the component–target network and protein-protein interaction (PPI) network, stigmasterol, mairin, sitosterol, and dehydrocostus lactone were identified as the key components, and RAC-alpha serine/threonine-protein kinase (AKT1), prostaglandin-endoperoxide synthase 2 (PTGS2), interleukin 1 beta (IL1B), caspase-3 (CASP3), and CASP8 were selected as the core targets. Gene ontology (GO) and Kyoto encyclopedia of genes and genomes (KEGG) enrichment results revealed the pharmacological mechanism of *Aucklandiae Radix* against gastric ulcer related to many biological processes and pathways, including antibacterial, anti-inflammatory, prostaglandin receptor response, and apoptosis. Molecular docking verification showed that the key components and core targets had good binding affinities. In the in vivo experiments, *Aucklandiae Radix* notably relieved the gastric ulcer by reducing the levels of tumor necrosis factor (TNF)-α, interleukin (IL)-1β, and myeloperoxidase (MPO) while improving the gastric histopathological features. *Conclusion*: The overall findings suggest that *Aucklandiae Radix* treats gastric ulcer with a multi-component, multi-target, and multi-mechanism model.

## 1. Introduction

Gastric ulcer (GU) is a high-incidence gastrointestinal disorder that can occur at any age, and about 10% of people worldwide suffer from GU [1]. When exposed to excessive acid and aggressive pepsin activity, ulcers occur on the gastric mucosa [2]. There are many causes of GU, including physical factors (excessive drinking), chemical factors (excessive use of non-steroidal anti-inflammatory drugs (NSAIDs), biological factors (Helicobacter pylori infection), and other factors (long-term mental tension, anxiety). These factors lead to the destruction of the balance between protective factors of the gastric mucosa (prostaglandins, gastric mucus, cell regeneration) and aggressive factors (gastric acid, pepsin secretion), resulting in the digestion of gastric mucosa by gastric acid and pepsin and thus causing ulcers [3,4]. In recent years, many Western drugs for the treatment of GU have been found, such as prostaglandin analogues, histamine receptor antagonists, proton pump inhibitors, and HP inhibitors, but they still have problems, including a high recurrence rate, and complications such as perforation, side effects of drugs, and bacterial resistance [5,6,7,8]. Therefore, the clinical treatment of GU urgently needs to find a more safe and reliable treatment.

In recent years, the use of plant-derived medicinal materials or extracts to treat GU has attracted the interest of researchers. *Aucklandia Radix* (AR), the dried root of Aucklandia lappa Decne, is a traditional herbal medicine in China. AR is warm in nature, and pungent and bitter in taste. Due to its effects of invigorating spleen and digesting food, promoting qi, and relieving pain, AR is widely used in the clinical treatment of digestive system diseases [9,10]. With the deepening of modern research, more than 200 chemical components of AR have been reported, including volatile oil, terpenoids, anthraquinones, and flavonoids [11]. These components have numerous pharmacological effects, such as antiviral [12], anti-inflammatory [13], antitumor [14], and promoting gastrointestinal movement [15,16]. The study showed that the extract of AR can significantly alleviate the gastric mucosal damage in rats with ethanol and pyloric ligation-induced acute gastric injury [15]. In addition, AR alcohol extract improves ethanol-induced gastric ulcer in rats by promoting the production of gastric mucus [16]. Therefore, from traditional to modern studies, AR and its prescriptions are well-used for treating gastritis, gastric ulcers, and other digestive system diseases.

Herbal medicine has the characteristics of being multi-component and multi-target in treating diseases. With the advancement of bioinformatics, the research strategy of a compound–target interaction such as network pharmacology and molecular docking has been developed to facilitate the study of phytomedicine with complicated mechanisms [17,18,19]. At present, although AR has a significant effect on the clinical treatment of gastric ulcers, there are few studies on its molecular mechanisms. In this study, we analyzed the potential active ingredients, action targets, and signaling pathways of AR in the treatment of GU from the level of molecular biology through a network pharmacology approach combined with in vivo experiments, and we verified them with molecular docking so as to provide a theoretical basis for the further study of the mechanism of AR on GU healing. 

## 2. Materials and Methods

### 2.1. Network Pharmacological Analysis

#### 2.1.1. Potential Active Components and Targets Prediction of AR

The chemical ingredients that met the conditions of oral bioavailability (OB) ≥30% and drug likeness (DL) ≥0.18 in the Traditional Chinese Medicine Systems Pharmacy Database and Analysis Platform (TCMSP, https://old.tcmsp-e.com, accessed on 25 July 2022) [20] were considered to be the active components of AR. In addition, because costunolide and dehydrocostus lactone are taken as index components of AR in the 2020 edition of the *Pharmacopoeia* of the People's Republic of China (ChP) [9], these two chemical components were also included in this study. Then, the targets of each active component were acquired from TCMSP, BATMAN-TCM (http://bionet.ncpsb.org.cn/batman-tcm/, accessed on 25 July 2022) [21] (score > 10), and SwissTargetPredition (http://www.swisstargetprediction.ch/, accessed on 25 July 2022) [22] databases; the unmatched genes in the UniProt database (https://www.uniprot.org/, accessed on 25 July 2022) were eliminated; the duplicate genes were removed; and finally, the potential targets of AR were obtained. 

#### 2.1.2. Disease Targets Prediction

Several databases including GeneCards (https://www.genecards.org/, accessed on 25 July 2022) [23], OMIM (https://omim.org/, accessed on 25 July 2022) [24], PharmGkb (https://www.pharmgkb.org/, accessed on 25 July 2022) [25], TTD (http://db.idrblab.net/ttd/, accessed on 25 July 2022) [26], and Drugbank (https://go.drugbank.com/, accessed on 25 July 2022) [27] were applied to screen the relevant targets related to “gastric ulcer” and remove those duplicate targets.

#### 2.1.3. Target of AR in the Treatment of GU

The common targets of AR and GU were obtained using Venny v2.1.0 (https://bioinfogp.cnb.csic.es/tools/venny/index.html, accessed on 25 July 2022), and a Venn diagram was drawn to display visually.

#### 2.1.4. Component–Target Network Construction

The interactions between drugs, components, targets, and diseases were analyzed through Cytoscape v3.8.0 software (San Diego, CA, USA) [28]. The degree value was calculated, and the component with a degree >10 was considered to be the key component of AR against GU.

#### 2.1.5. PPI Network Construction

The shared targets were retrieved using STRING (https://cn.string-db.org/, accessed on 27 July 2022) [29], and the Tab-separated values (TSV) format file of protein interaction results was obtained. Then, the protein–protein interaction (PPI) interaction network was visualized by the Cytoscape v3.8.0 software, and the number of connections between each node in the network was expressed by a degree value. Subsequently, the top 10 targets of degree values were considered to be the main action targets.

#### 2.1.6. GO and KEGG Pathway Enrichment Analysis

In order to deeply understand the function of the previously obtained common targets of AR for the treatment of GU, the Metascape database (https://metascape.org/gp/index.html#/main/step1, accessed on 27 July 2022) [30] was used to analyze the Gene Ontology (GO) and Kyoto Encyclopedia of Genes and Genomes (KEGG) pathways. The results were sorted and visualized according to the enriched *p*-value.

### 2.2. Molecular Docking

The 2D structure of the compound was obtained from the PubChem database (https://pubchem.ncbi.nlm.nih.gov/, accessed on 27 July 2022), optimized by ChemBio3D Ultra 14.0.0.117 software (Waltham, MA, USA), and converted to PDB format. The crystal structures of targets were acquired from the RSCB PDB database (https://www.rcsb.org/, accessed on 28 July 2022) [31]. Compounds and targets were converted from their native formats into pdbqt formats with AutoDockTools [32]. The structures were optimized by deleting water molecules and adding hydrogen atoms. Then, the molecular docking study was performed using Autodock Vina. The coordinates of the target active pocket are listed in Table 1. Size_x = 60, Size_y = 60, and Size_z = 60 in each target. All docking run options were set to default values. Finally, the docking results with the highest scores were visualized by PyMoL.

### 2.3. In Vivo Experiment

#### 2.3.1. Materials and Reagents

The dried root of AR materials (batch No.: 2205101) was obtained from Hainan Shounanshan Ginseng Industry Co., Ltd. (Hainan, China). Identification of the AR was performed by an expert from the Identification Teaching and Research Office of Hainan Medical University. Indomethacin was obtained from Sigma Chemical Co. (St. Louis, MO, USA), (Lot# 088M4033V). Ranitidine (batch No.: 2101532) was bought from Foshan Chiral Pharmaceutical Co., Ltd. (Foshan, China). Rat tumor necrosis factor-α (TNF-α, catalog No.: JM-01587R1), interleukin-1β (IL-1β, catalog No.: JM-01454R1), myeloperoxidase (MPO, catalog No.: JM-01744R1), and Prostaglandin E2 (PGE2, catalog No.: JM-01475R1) enzyme-linked immunosorbent assay (ELISA) kits were obtained from Jingmei Biotechnology Co. (Taixing, China), Ltd. Hematoxylin-eosin (H&E) staining kits were purchased from the Nanjing Jiancheng Bioengineering Institute (Nanjing, China).

#### 2.3.2. Preparation of AR Extract

The AR ethanol extract was prepared using traditional methods. Briefly, 500 g of material was extracted with 5 volumes of (1:5, *w*/*v*) 95% ethanol at reflux for 2 h twice, then we combined the filtrate, volatilized the ethanol at a low temperature, and concentrated in vacuum to obtain the final freeze-dried AR extract (ARE). The yield was 10.26%.

#### 2.3.3. Animals and Treatment

Thirty-six male SD rats (weighing 200–220 g) were obtained from the Changsheng Biotechnology Company (Changchun, China). Animal experimental procedures were reviewed and approved by the Ethics Committee of Inner Mongolia Minzu University (Ethics Number: NM-LL-2021-06-15-1, approved on 15 June 2021). All animals were housed in standard laboratory conditions (constant temperature of 24.0 ± 2.0 °C, relative humidity of 50 ± 5%, 12 h light/dark cycle), and allowed free access to food and water. The animals were acclimatized for 1 week before conducting the formal experiments.

The animals were divided randomly into 6 groups (*n* = 6): the control group (Carboxymethyl cellulose sodium, CMC-Na), the model group (30 mg/kg dose indomethacin only), the ranitidine group (40 mg/kg dose ranitidine), and the ARE group (0.15, 0.3, and 0.6 g/kg dose) according to the clinically recommended dose [33]. All substances used were dissolved in 0.5% CMC-Na and administered to rats intragastrically once per day for two consecutive weeks. On the 13th day, rats were stripped of food but allowed to drink water freely for 24 h. On day 14, a single oral dose of indomethacin (30 mg/kg bw., suspended in 0.5% CMC-Na) was administered to all groups except the control group to establish a GU model. Six hours later, all the rats were euthanized with an overdose of anesthesia. The gastric tissue of rats was separated carefully, cut along the great curvature, and flushed with cold saline. The ulcer area (mm^2^) of gastric mucosa was measured by ImageJ-win64 software (Bethesda, MD, USA). 

#### 2.3.4. H&E Staining

The gastric tissue samples were fixed with 4% paraformaldehyde, dehydrated by gradient ethanol, cleared in xylene, and embedded in paraffin. Then, the processed tissue was sliced into 5.0 μm thickness and stained with the H&E solution. Pathological changes in the gastric tissues were observed with a light microscope.

#### 2.3.5. Determination of Biochemical Indices of Gastric Tissue 

**Figure 1:**
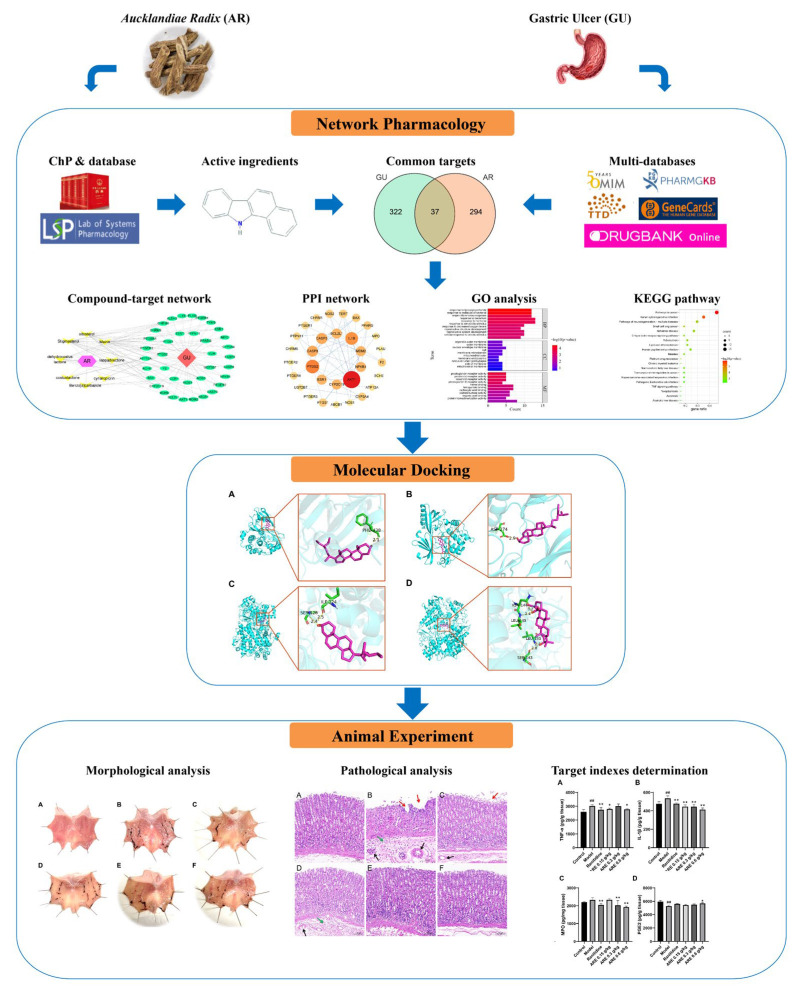
Workflow of this study. In the molecular docking section, (**A**) The docking result of AKT1 with stigmasterol, (**B**) The docking result of AKT1 with sitosterol, (**C**) The docking result of PTGS2 with stigmasterol, (**D**) The docking result of PTGS2 with mairin; In morphological and pathological section, (**A**) Control group, (**B**) Model group, (**C**) Ranitidine group, (**D**) ARE 0.15 g/kg group, (**E**) ARE 0.3 g/kg group, (**F**) ARE 0.6 g/kg group; the red arrow, epithelial cell loss; the green arrow, inflammatory exudation and infiltration; and the black arrow, vasocongestion; In target indexes determination section, (**A**) TNF-α, (**B**) IL-1β, (**C**) MPO, (**D**) PGE2; ## *p* < 0.01 when compared with the control group; * *p* < 0.05, ** *p* < 0.01 when compared with the model group.

The collected stomach tissues were cut and weighed, the corresponding volume of PBS (1 g: 9 mL) solution was added, then ground on ice, and the mixture was centrifuged at 5000 rpm for 10 min to collect the gastric tissue homogenate. The levels of TNF-α, IL-1β, MPO, and PGE2 in the gastric tissue were determined by commercial assay kits according to the instructions. The overall workflow is shown in Figure 1.

### 2.4. Statistical Analysis

All data were expressed as mean ± standard error of mean (S.E.M.) of the parameters. Statistical significances between the groups were analyzed by one-way ANOVA with the uncorrected Fisher’s Least-SignificantDifference (LSD) test using GraphPad Prism 8.0.2 software (San Diego, CA, USA). Statistical significance was expressed by * *p* < 0.05 or ** *p* < 0.01.

## 3. Results

### 3.1. Prediction of Potential Bioactive Components and Targets of AR

In the TCMSP database, six components of AR that accorded with the conditions of OB ≥ 30% and DL ≥ 0.18 were obtained. Costunolide (OB = 60.48, DL = 0.11) and dehydrocostus lactone (OB = 58.57, DL = 0.14) were included together according to ChP though they did not meet the inclusion criteria. Taken together, a total of eight potential bioactive ingredients were used for the subsequent analysis, as shown in Table 2. Then, the corresponding targets of these potential active ingredients were searched in the TCMSP, BATMAN-TCM, and SwissTargetPredition databases, and 49, 125, and 197 targets were found, respectively. Finally, 331 targets for AR were collected after deleting the duplicates.

### 3.2. Target of AR in the Treatment of GU

**Figure 2:**
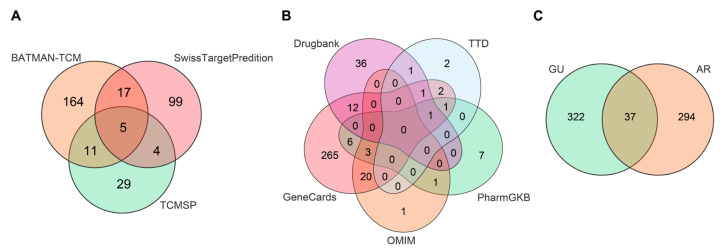
Target screening involved in AR for the treatment of GU. (**A**) Venn diagram of potential targets of AR. (**B**) Venn diagram of disease targets. (**C**) Venn diagram of potential targets of AR for the treatment of GU.

In total, three hundred and eleven, ninety-seven, nineteen, nine, and seventy-nine known disease targets related to GU were obtained from GeneCards, OMIM, PharmGKB, TTD, and Drugbank, respectively. After merging these targets and removing duplicates, a total of 359 GU targets were collected. Then, the 331 targets of AR and the 359 targets of GU were intersected, and 37 common targets were obtained, which were the potential targets of AR against GU (Figure 2).

### 3.3. Bioactive Component–Target Network of AR against GU

**Figure 3:**
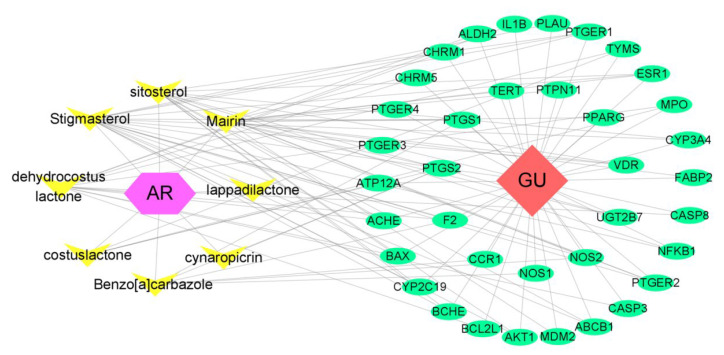
Compound–disease–target network. The purple hexagon represents AR. The yellow triangle represents the active compound contained in AR. The red diamond represents GU. The green ellipse represents the targets.

A compound–target network was constructed to clarify how the bioactive compounds of AR may act against GU. There were 47 nodes and 114 edges in the compound–target network, and the results are shown in Figure 3. According to the topological analysis, stigmasterol (degree = 19), mairin (degree = 16), sitosterol (degree = 15), and dehydrocostus lactone (degree = 12) were predicted to be the key components for the treatment of GU.

### 3.4. PPI Network Construction and Hub Targets

**Figure 4:**
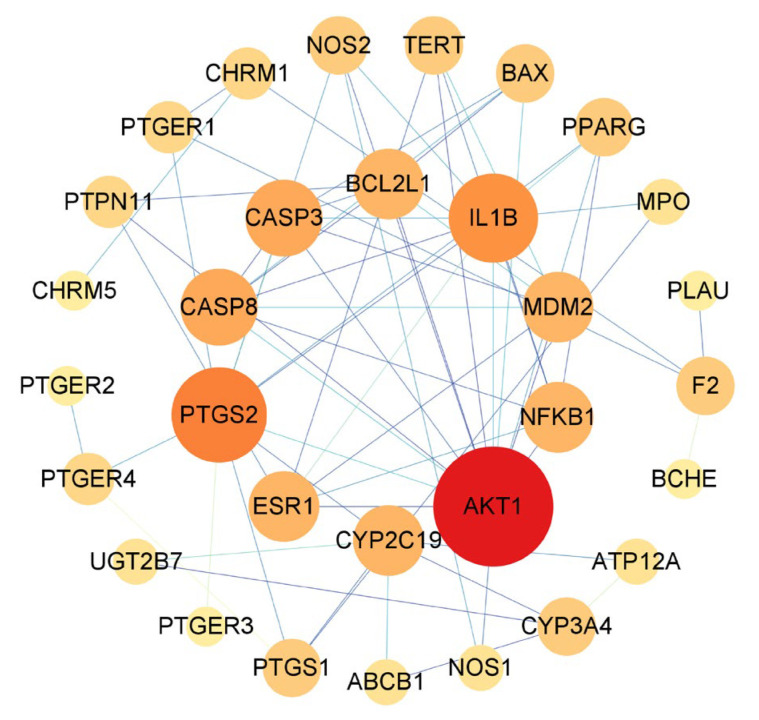
The PPI analysis of targets between AR and GU. The node size and color are proportional to the target degree in the network.

The common targets of AR and GU were uploaded to the STRING database, and the PPI network was visualized through Cytoscape 3.8.0 software. There were 31 nodes (six free nodes were hidden) and 66 edges in the network. The top 10 key targets were screened according to the degree value: AKT1, PTGS2, IL1B, CASP3, CASP8, CYP2C19, NFKB1, MDM2, BCL2L1, and ESR1, which were speculated to be the core targets of AR for the treatment of GU (Table 3 and Figure 4).

### 3.5. GO and KEGG Pathway Enrichment Analysis

**Figure 5:**
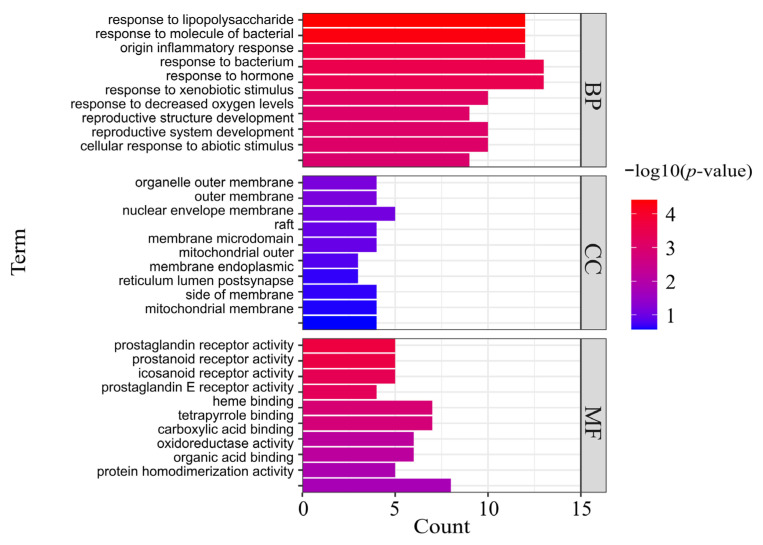
Top 10 significantly enriched terms in biological process (BP), cellular component (CC), and molecular function (MF) of GO analysis. The y-axis represents the GO term. The x-axis represents the gene number, and the color represents the *p*-value.

**Figure 6:**
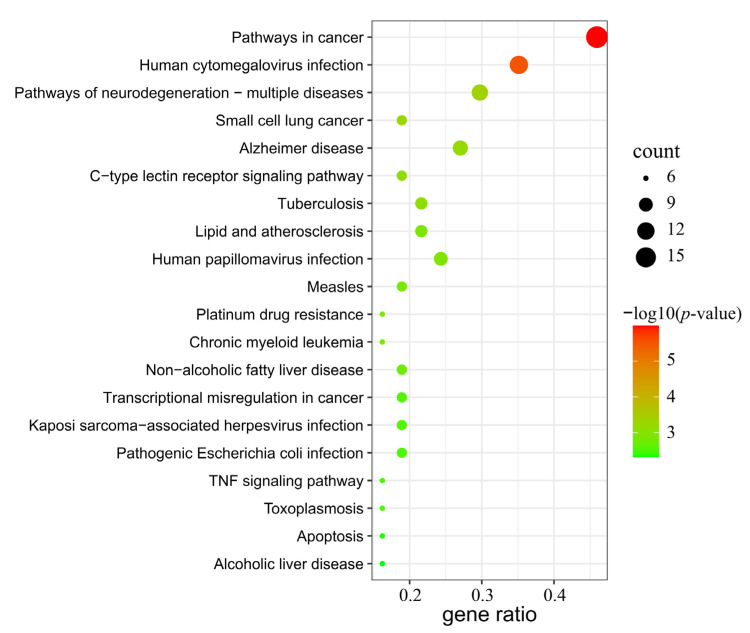
The top 20 significantly enriched pathways of the candidate targets of AR in the treatment of GU. The y-axis represents the KEGG pathway. The x-axis represents the ratio of genes enriched in the pathway. The size of bubbles indicates the number of targets, and the color indicates the *p*-value.

The common targets of AR and GU were uploaded to the Metascape platform to analyze GO and KEGG pathways, and a total of 491 GO entries and 102 KEGG pathways were obtained. A bar diagram (Figure 5) and bubble diagram (Figure 6) were drawn, respectively, according to the p-value ranking. The findings of GO enrichment suggested that these common targets were associated with biological activities such as response to lipopolysaccharide, inflammatory response, response to bacterium, and response to hormone; cell components such as organelle outer membrane, outer membrane, nuclear envelope; and molecular functions such as prostaglandin receptor activity, heme binding, and organic acid binding. These items were closely related to gastric mucosal injury and repair. The KEGG enrichment results showed that the shared targets were significantly enriched in pathways including pathways in cancer, human cytomegalovirus infection, pathways of neurodegeneration, the C-type lectin receptor signaling pathway, transcriptional misregulation in cancer, the TNF signaling pathway, apoptosis, etc. These pathways were related to gastric mucosal injury, ulcer healing, and ulcer development to gastric cancer. The above results indicated that the targets of the main bioactive components of AR were distributed in different signaling pathways.

### 3.6. Molecular Docking Verification

**Figure 7:**
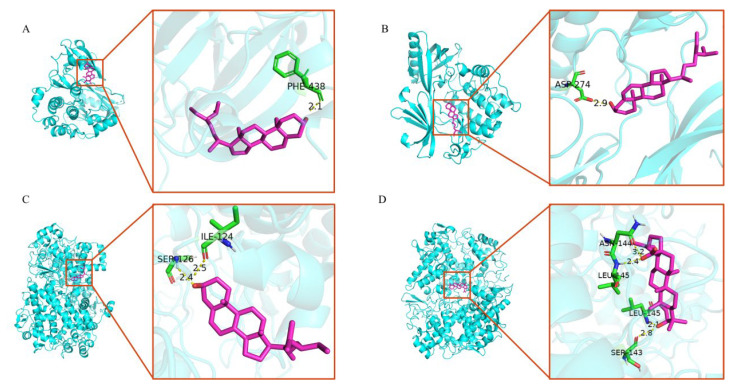
The 3D interaction diagrams of AR core targets against GU. (**A**) The docking result of AKT1 with stigmasterol. (**B**) The docking result of AKT1 with sitosterol. (**C**) The docking result of PTGS2 with stigmasterol. (**D**) The docking result of PTGS2 with mairin. The purple sticks indicate the ligands, the green sticks indicate the active site residues of targets, and the hydrogen bonds are indicated by yellow dotted lines.

In addition, molecular docking was performed to elucidate the interactions between the key targets obtained from the PPI analysis and the main bioactive components obtained from the network analysis. The docking results showed that these main components were combined well with the active site residues of key targets. Table 4 shows the minimum binding energy of each docking module. As shown in Figure 7, one hydrogen bond was formed between stigmasterol and AKT1 (Figure 7A), and one hydrogen bond was formed between sitosterol and AKT1 (Figure 7B). Furthermore, stigmasterol formed two hydrogen bonds with Ile124 and Ser126 in PTGS2 (Figure 7C); mairin formed two hydrogen bonds with Leu145 and Ser143 in the A chain of PTGS2, and it formed two hydrogen bonds with Asn144 and Leu145 in the B chain of PTGS2 (Figure 7D). The results indicated that the components could bind to the active sites of the targets.

### 3.7. Effect of AR Extract on Indomethacin-Induced GU

**Figure 8:**
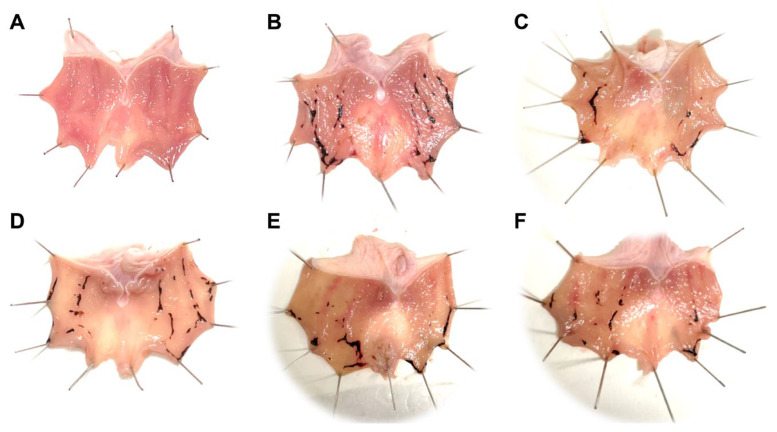
Results of gastric morphology. (**A**) Control group. (**B**) Model group. (**C**) Ranitidine group. (**D**) ARE 0.15 g/kg group. (**E**) ARE 0.3 g/kg group. (**F**) ARE 0.6 g/kg group. ARE, Aucklandia Radix ethanol extract.

As can be seen from the gastric morphology in Figure 8, the rats in the control group displayed no damage to the mucosal. However, the stomachs of indomethacin-treated rats appeared to have severe mucosal erosion, liner and punctate bleeding compared to the rats in the control group, implying that the GU model was successfully established. In contrast, animals pre-treated with ranitidine or ARE showed notably fewer gastric lesions in comparison to the model group.

The quantitative analysis of the ulcer area was consistent with the morphological observations (Table 5). Compared with the model group, the ulcer areas in the ranitidine and ARE 0.6 g/kg groups were reduced obviously (*p* < 0.05). Among the three dose groups of ARE, the ARE 0.6 g/kg group had the lowest ulcer area along with the highest inhibition rate (50.26%, *p* < 0.05).

**Figure 9:**
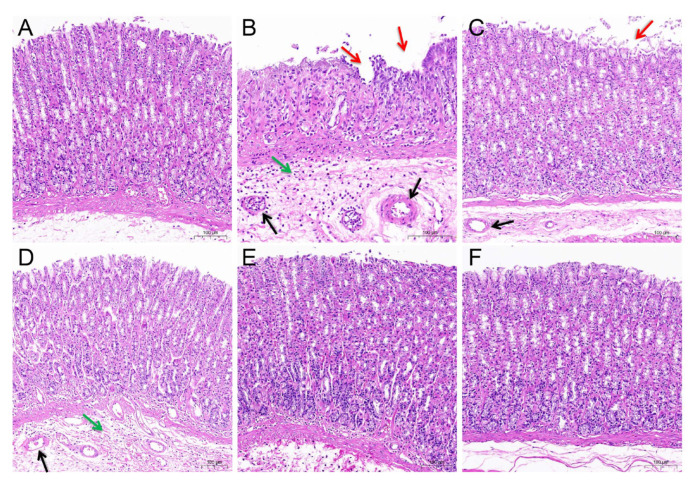
Results of gastric H&E section. (**A**) Control group. (**B**) Model group. (**C**) Ranitidine group. (**D**) ARE 0.15 g/kg group. (**E**) ARE 0.3 g/kg group. (**F**) ARE 0.6 g/kg group. ARE, Aucklandia Radix ethanol extract. The red arrow indicates epithelial cell loss, the green arrow indicates inflammatory exudation and infiltration, and the black arrow indicates vasocongestion.

Then, H&E staining was used to investigate the protective effects of ARE against GU (Figure 9). In the control group, the gastric mucosa showed a normal histological overview. However, the administration of indomethacin caused a series of gastric lesions including the loss of epithelial cells, inflammatory exudation and infiltration, and vascular congestion. Pre-treatment with ranitidine and ARE (0.15, 0.3, 0.6 g/kg) showed significantly attenuated indomethacin-induced pathological deterioration.

### 3.8. AR Extract Alleviated Indomethacin-Induced Inflammation and Oxidative Stress Damage

**Figure 10:**
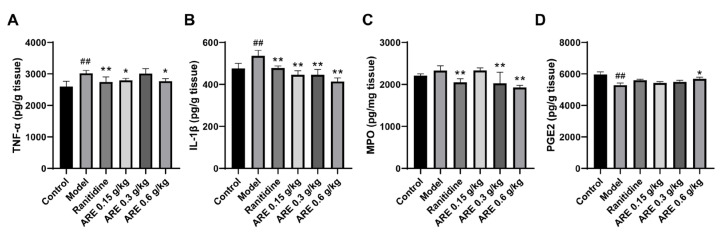
The effects of ARE on TNF-α, IL-1β, MPO, and PGE2 in gastric tissue. (**A**) TNF-α. (**B**) IL-1β. (**C**) MPO. (**D**) PGE2. ## *p* < 0.01 when compared with the control group; * *p* < 0.05, ** *p* < 0.01 when compared with the model group.

Subsequently, the levels of TNF-α, IL-1β, MPO, and PGE2 in the gastric tissue were detected. The results showed that indomethacin increased the secretion of TNF-α (*p* < 0.01) and IL-1β (*p* < 0.01) and MPO, whereas it reduced the secretion of PGE2 in gastric tissues compared to the control group. Notably, the pre-treatment with ranitidine and ARE reversed the above alterations (Figure 10).

## 4. Discussion

AR is a traditional herbal medicine with gastrointestinal protective effects. In this study, network pharmacology combined with an in vivo experiment were used to explore the potential material foundation, action targets, and possible molecular mechanisms of AR on the treatment of GU in order to explain the gastric protective effect of AR components. As a result, four main bioactive components (stigmasterol, mairin, sitosterol, and dehydrocostus lactone) and 10 core targets (AKT1, PTGS2, IL1B, CASP3, CASP8, CYP2C19, NFKB1, MDM2, BCL2L1, and ESR1) of AR against GU were predicted through the component–target network and PPI analysis. Molecular docking simulations indicated that the predicted main bioactive ingredients and core targets had good binding affinity. The enrichment analysis of GO and KEGG revealed that the protective role of AR against GU involved various GU healing-related biological processes and signaling pathways. Meanwhile, the in vivo experiments showed that the ethanol extract of AR alleviated the GU induced by indomethacin, which was attributed to the regulatory effects of TNF-α, IL-1B, MPO, and PGE2.

Medical plants have significant effects in the treatment of GU, which depend on their multiple chemical components. Therefore, it is particularly important to reveal the chemical components of natural plants. At present, the anti-ulcer effect and mechanism of many kinds of medicinal plants and their components have been revealed. For example, previous reports have recorded that the aged garlic and garlic oil extract enhanced the expression of PGE2, thus protecting the stomach injury caused by indomethacin and sodium taurocholate, and this effect may be related to the fatty acids and flavonoids abundant in garlic [34,35]. However, further exploration is needed to clarify which ingredient is most effective. The network pharmacology method provides convenience for predicting and revealing the effective components of medicinal plants. The anti-ulcer potential or gastro-protective effects of the main bioactive components predicted in this study have been reported in previous research. Sitosterol and stigmasterol have protective effects on gastroduodenal mucosa [36]. For example, the study by Zhao et al. showed that stigmasterol could induce apoptosis and protective autophagy of gastric cancer cells through inhibiting the Akt/mTOR pathway [37]. Onwuchekwa et al. reported that using 1.5 and 3.0 mg/kg mairin (betulinic acid) could inhibit indomethacin-induced GU and could also significantly promote the secretion of gastric mucus [38]. Dehydrocostus lactone could relieve ethanol-induced gastric ulcers in mice, which was considered to be related to the decrease in TNF-α, COX-2, and MDA and the increase in IL-10 and PCNA [39]. Therefore, the core components and targets predicted by network pharmacology are persuasive.

As an important part of the digestive system, the stomach has many special microbial community structures [40]. Gastrointestinal flora, especially Helicobacter pylori, play a crucial role in the pathogenesis of GU [41]. At present, many key antibacterial components have been screened for AR. For example, costuslactone and dehydrocostus lactone can inhibit Helicobacter pylori [42] and Streptococcus mutans [43], and sitosterol can reduce the survival rate of Salmonella typhimurium in cells [44]. Another study found that dehydrocostus lactone can promote the polarization of M2 macrophages and reduce M1 polarization by regulating MAPK/NF-kB and AMPK/Nrf2 pathways, thus reducing the inflammatory reaction caused by Gram-positive bacteria [45]. Our GO enrichment analysis results also showed that the effect of AR against GU involved the responses to lipopolysaccharide, response to a molecule of bacterial origin, and response to bacterium, indicating that AR could reduce the damage of harmful factors to the gastric mucosa caused by Helicobacter pylori and other bacteria. In addition, according to the KEGG pathway enrichment results, the human cytomegalovirus infection pathway was related to the anti-ulcer effect of AR, and studies have shown that cytomegalovirus virus infection can cause GU [46], suggesting that AR can be used to treat GU caused by the cytomegalovirus virus.

When the stomach is infected by Helicobacter pylori, stimulated by ethanol or NSAIDs, neutrophils and monocytes may infiltrate the gastric mucosa, producing inflammatory cytokines such as TNF-α, IL-6, and IL-1β, which are the important factors causing gastric mucosal damage [47]. IL-1β is a key proinflammatory cytokine that is essential for cell defense and tissue repair in almost all tissues. IL-1β has many biological functions, which are closely related to pain, inflammation, and autoimmunity, and is involved in neuroprotection, tissue remodeling and repair, and the regulation of IL-6 and TNF-α, etc. [48]. We found that IL-1β is one of the core targets of AR against GU and has good binding ability with the dehydrocostus lactone (Table 4). Existing studies have shown that the selected components can reduce the damage of the disease by downregulating the expression of IL-1β, which confirms its strong anti-inflammatory activity. For example, cynaropicrin inhibited renal tissue damage caused by acute renal injury in septic rats by downregulating IL-1β [49]; Costuslactone and dehydrocostus lactone significantly decreased the mRNA level of IL-1β in RAW264.7 cells stimulated with lipopolysaccharide (LPS) [50]. In addition, our GO and KEGG enrichment results showed that the common targets of AR and GU were significantly enriched in the inflammatory response, TNF, NF-kB, and IL-17 signaling pathway. Studies have shown that these biological functions and pathways can regulate proinflammatory factors to alleviate GU [51,52]. Our findings support this speculation. According to the animal experiments, ARE restored the elevated TNF-α and IL-1β contents caused by indomethacin (Figure 10A,B), which verified that AR improves GU by having an anti-inflammatory effect.

Prostaglandin (PG) is an important mucosal defense and repair mediator in the gastrointestinal tract, among which PGE2 is the most effective one. Cyclooxygenase (COX) is the key rate-limiting enzyme in PG synthesis [53]. COX has two isoenzyme types: COX-1 and COX-2. COX-1 is encoded by the PTGS1 gene and is rich in the gastrointestinal tract, producing PG to play a protective role in the gastrointestinal tract; COX-2 is encoded by the PTGS2 gene and expressed at a low level in the stomach, but it will be rapidly induced by inflammatory stimulation or injury, which will promote the gradual expansion of inflammation and also inhibit the expression of COX-1 and reduce the PG synthesis, thereby weakening the gastric mucosal defense function [54]. Zheng et al. found that costuslactone can inhibit the expression of COX-2 in mice with ethanol-induced GU, showing gastric protection [39]. According to Bi et al. β-sitosterol can regulate MAPKs and the NF-κB signaling pathway and decrease the expression of TNF-α, COX-2, and IL-6 [55]. Liang et al. reported that stigmasterol alleviated cerebral ischemia/reperfusion injury by downregulating the expression of COX2 and NF-kB [56]. In our study, PTGS2 was one of the core targets of AR against GU and had a good binding affinity with the main bioactive components (stigmasterol and mairin) of AR. In addition, PGE2 has four specific subtypes of G protein-coupled receptors (GPCRs), EP1-EP4, which are closely related to gastric contraction, gastric mucus secretion, angiogenesis, gastric injury healing, etc. [53]. According to our GO enrichment results, the common targets of AR and GU were significantly enriched in the biological process of prostaglandin receptor activity, which involved EP1-EP4 (PTGER1-4). According to a study by Kim et al. PTGER2 and PTGER3, which play an inhibitory role in gastrin or gastric acid secretion, were directly related to gastric cancer [57]. Heinrichs et al. verified that the upregulation of PTGER4 expression in gastric tissue was a risk factor for the pathogenesis of gastric cancer [58]. Furthermore, our in vivo experiment also implied that the level of PGE2 was downregulated in GU rats and upregulated in the ARE-treated group, indicating that AR may regulate the content of PGE2 to inhibit the GU (Figure 10D). Therefore, it can be inferred from these results that AR may improve GU and even prevent it from developing into gastric cancer by regulating COX2/PTGS2 and PGE2.

Furthermore, the balance between the proliferation and apoptosis of gastric epithelial cells is crucial for the integrity of gastric mucosa. Gastric ulcer will occur when various pathogenic factors disrupt this balance [59]. Studies have shown that the main active components of AR can promote apoptosis. Cheng et al. found that the derivatives of mairin (betulinic acid) and oleanolic acid triggered the apoptosis of three human cancer cell lines through ROS-mediated activation of the caspase-3 signaling pathway [60]. Oh et al. showed that dehydrocostus lactone could induce the apoptosis of human leukemia HL-60 cells through enhancing caspase-3 and caspase-8 [61]. Stigmasterol inhibits the proliferation of gastric cancer cells by inhibiting the Akt/mTOR signaling pathway, inducing apoptosis and protective autophagy [37]. It was also reported that stigmasterol can inhibit apoptotic responses through decreasing Bax and CASP3 expression and increasing Bcl-Xl expression [56]. In addition, cynaropicrin inhibited the G2/M cycle and induced apoptosis in gastric adenocarcinoma AGS cells [62]. According to the PPI analysis, AKT1, CASP3, CASP8, BCL2L1, MDM2, and NFKB1 were closely related to the regulation of cell cycle arrest and apoptosis. Our KEGG enrichment results showed that the shared targets of AR and GU were significantly enriched in the apoptotic pathway.

In summary, the present study analyzed the potential active components and mechanism of AR in the treatment of GU through network pharmacology, and revealed the multi-component, multi-target, and multi-pathway anti-ulcer mode of AR; in vivo experiments also showed that the AR extract can prevent indomethacin-induced GU, and its mechanism is related to the regulation of TNF-α, IL-1B, and PGE2. These findings provide important evidence for future research into the mechanism of AR against GU, and also provide an experimental basis for the further development and utilization of AR. However, due to the accuracy, integrity, and reliability of the database used in network pharmacology, the potential active ingredients and core targets obtained in this study may be different or missing. Therefore, in future experiments, we still need to further confirm the mechanism of these active ingredients against GU through a number of clinical trials and in vivo experiments.

## 5. Conclusions

To sum up, AR treats GU through a multi-component, multi-target, and multi-mechanism mode: stigmasterol, mairin, sitosterol, dehydrocostus lactone, and other active compounds may exert anti-ulcer effects through AKT1, PTGS2, IL1B, CASP3, CASP8, and other targets, which are mainly related to mechanisms including the inhibition of bacteria and viruses, anti-inflammatory activity, regulation of PGE2, regulation of cell proliferation and apoptosis, and other pathways. Meanwhile, AR can prevent the damage of gastric mucosa induced by indomethacin and promote ulcer healing through reducing TNF-α, IL-1β, and MPO, and increasing the PGE2 in the gastric tissue. AR may be an effective multi-target anti-GU herbal drug.

## Figures and Tables

**Table 1:** Parameters of molecular docking between AR and targets.

Target	center_x	center_y	center_z
AKT1	26.172	−14.865	−7.993
PTGS2	22.594	40.999	39.56
IL1B	38.283	13.249	68.618
CASP3	26.814	22.732	38.207
CASP8	55.527	72.326	135.502

**Table 2:** Potential active ingredients of Aucklandiae Radix (AR).

Mol ID	Molecule Name	OB (%)	DL	PubChem ID	2D Structure
MOL010813	Benzo[a]carbazole	35.22	0.22	9196	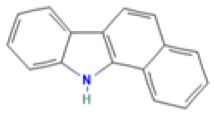
MOL010828	cynaropicrin	67.5	0.38	119093	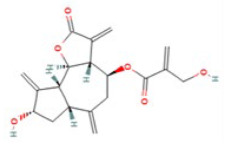
MOL010839	lappadilactone	38.56	0.73	103581781	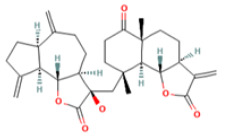
MOL000211	Mairin	55.38	0.78	64971	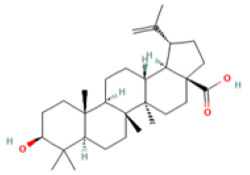
MOL000359	sitosterol	36.91	0.75	222284	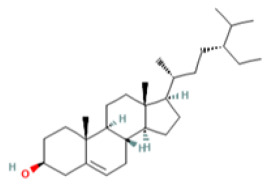
MOL000449	Stigmasterol	43.83	0.76	5280794	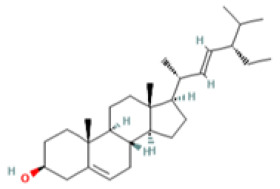
MOL001298	dehydrocostus lactone	58.57	0.14	73174	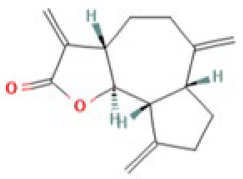
MOL010826	costuslactone	60.48	0.11	162416062	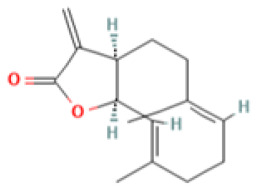

**Table 3:** The potential hub proteins ranked as top 10.

No.	Uniprot ID	Gene Name	Protein Name	Degree
1	P31749	AKT1	RAC-alpha serine/threonine-protein kinase	14
2	P35354	PTGS2	Prostaglandin G/H synthase 2	10
3	P01584	IL1B	Interleukin-1 beta	9
4	P42574	CASP3	Caspase-3	7
5	Q14790	CASP8	Caspase-8	7
6	P33261	CYP2C19	Cytochrome P450 2C19	6
7	P19838	NFKB1	Nuclear factor NF-kappa-B p105 subunit	6
8	Q00987	MDM2	E3 ubiquitin-protein ligase Mdm2	6
9	Q07817	BCL2L1	Bcl-2-like protein 1	6
10	P03372	ESR1	Estrogen receptor	6

**Table 4:** Binding energy between the key targets and main ingredients.

Key Targets (PDB ID)	Stigmasterol	Mairin	Sitosterol	Dehydrocostus Lactone
AKT1 (7NH5)	−10.4	-	−9.9	-
PTGS2 (5F19)	−9.3	−9.4	-	-
IL1B (5R8Q)	-	-	-	−6.9
CASP3 (2DKO)	-	-	-	−6.4
CASP8 (4JJ7)	-	-	-	−7.7

**Table 5:** Effects of ARE against indomethacin-induced GU.

Group	Ulcer Area (mm^2^)	Inhibition Rate (%)
Control	0	-
Model	45.0 ± 17.6 ##	-
Ranitidine 40 mg/kg	17.5 ± 4.8 *	61.11
ARE 0.15 g/kg	36.6 ± 3.8	18.61
ARE 0.3 g/kg	30.8 ± 6.2	31.62
ARE 0.6 g/kg	22.4 ± 4.3 *	50.26

Notes: ARE, Aucklandia Radix ethanol extract; (##) represents the significant differences compared to the control group at *p* < 0.01, whereas (*) represents the significant differences compared to the model group at *p* < 0.05.

## Data Availability

Not applicable.

## Abbreviations

GU, gastric ulcer; NSAIDs, non-steroidal anti-inflammatory drugs; AR, Aucklandia Radix; OB, oral bioavailability; DL, drug likeness; TCMSP, Traditional Chinese Medicine Systems Pharmacy Database and Analysis Platform; ChP, Pharmacopoeia of the People’s Republic of China; PPI, protein–protein interaction; GO, gene ontology; KEGG, Kyoto Encyclopedia of Genes and Genomes; TNF-α, tumor necrosis factor-α; IL-1β, interleukin-1β; MPO, myeloperoxidase; PGE2, Prostaglandin E2; ELISA, enzyme-linked immunosorbent assay; H&E, Hematoxylin-eosin; ARE, Aucklandia Radix extract; CMC-Na, Carboxymethyl cellulose sodium; S.E.M, standard error of mean; PG, prostaglandin; COX, cyclooxygenase; GPCRs, G protein-coupled receptors.
